# Comparison of pre-diagnostic signs and symptoms of young-onset dementia and late-onset dementia in the general practice

**DOI:** 10.1177/13872877261451197

**Published:** 2026-05-27

**Authors:** Caitlin Hibbs, Stevie Hendriks, Jean Muris, Ralph Leijenaar, Dorothee Horstkötter, Sebastian Köhler, Marjolein De Vugt

**Affiliations:** 1 Department of Psychiatry & Neuropsychology, Mental Health and Neuroscience Research Institute, Alzheimer Centre Limburg, 5211 Maastricht University, Maastricht, The Netherlands; 2 Department of Family Medicine, Care and Public Health Research Institute, 5211 Maastricht University, Maastricht, The Netherlands; 3 Department of Health, Ethics and Society, Mental Health and Neuroscience Research Institute, 5211 Maastricht University, Maastricht, The Netherlands

**Keywords:** Alzheimer's disease, pre-diagnostic trajectory, symptoms, young-onset dementia

## Abstract

**Background:**

Dementia can occur in older adults (late-onset dementia; LOD) or before age 65 (young-onset dementia; YOD). YOD often involves more heterogeneous subtypes and atypical early symptoms, making it harder for general practitioners (GPs) to recognize YOD early.

**Objective:**

To identify differences in pre-diagnostic signs and symptoms between persons with YOD and LOD at the GP, enabling earlier recognition of YOD.

**Methods:**

This case-control study used data from GP records of 88 YOD cases and 174 LOD controls from the Research Network Family Medicine database, matched by sex and GP practice. Persons diagnosed before age 70 were included in the YOD group to account for diagnostic delay. Symptoms up to five years before diagnosis were grouped into eight categories. Differences in symptom presence were analyzed per year using binary logistic regression.

**Results:**

Compared to LOD, YOD cases had significantly higher odds of reporting affective symptoms two (OR = 2.43, 95%CI = 1.30–4.51) and one year (OR = 2.36, 95%CI = 1.35–4.13) before diagnosis. Social indicators were also more common in YOD five (OR = 4.84, 95%CI = 1.09–21.44), four (OR = 3.75, 95%CI = 1.27–11.09), and one (OR = 4.00, 95%CI = 2.33–6.86) year before diagnosis. Gait disturbances were less frequent in YOD at four (OR = 0.33, 95%CI = 0.12–0.89) and two (OR = 0.43, 95%CI = 0.20–0.91) years prior. Surprisingly, no significant differences emerged for cognitive symptoms.

**Conclusions:**

YOD differs from LOD in several symptom categories up to five years before diagnosis. These findings provide insight into the pre-diagnostic trajectory of YOD and may support earlier recognition.

## Introduction

Currently, approximately 55 million people are living with different types of dementia worldwide. This number is expected to increase to 78 million in 2030 and 139 million in 2050.^1^ A recent study has shown that 3.9 million people have young-onset dementia (YOD),^2^ which refers to the onset of dementia symptoms before the age of 65 years.^3^

When diagnosed with YOD, people often still fulfil multiple social roles, such as being a parent of younger children, employee, or caregiver.^4^ The diagnosis can have a significant psychosocial impact on both the person with dementia and their family.^5^ Job loss due to diagnosis can lead to financial difficulties,^6,7^ family relationships shift to the role of caregiver, and lack of understanding from others can lead to social isolation.^8^

Still, dementia is commonly perceived as a disorder of older age (late-onset dementia, LOD) and to primarily involve symptoms such as forgetfulness and spatial or temporal disorientation. Early warning signs of LOD include memory loss, confusion, misplacing items and poor judgment.^9^ However, people with YOD tend to experience different early symptoms. YOD typically has an insidious onset with initial symptoms often overlooked or misattributed to routine stress or tension, fatigue, or psychiatric disorders.^5^ We previously showed that first symptoms of YOD may include cognitive impairment up to 5 years prior to diagnosis, but behavioral changes, personality changes, language, visual or motor problems are also common.^10^ Compared to LOD, YOD is caused by a much wider range of subtypes of dementia, all with different first symptoms.^11^ Alzheimer's disease (AD) is the leading cause of dementia in both YOD and LOD, but the proportions differ significantly: AD accounts for about 34% of YOD cases and around 60–70% of LOD cases.^1,12^ Other common YOD subtypes include vascular dementia (about 20%), frontotemporal dementia (about 12%), and Lewy body dementia (about 10%).^13^ The most common symptoms are memory impairment, impaired judgment and problem-solving abilities, language problems, and visuospatial dysfunction, depending on the subtype.^14,15^ Furthermore, even within the same dementia subtype, persons with YOD and LOD can have differing clinical presentations. A previous study demonstrated that non-memory presentations are five times more frequent in early-onset AD than in late-onset AD, with individuals in the former experiencing fewer memory impairments but more visuospatial dysfunction and language problems.^16^ Additionally, another study revealed that persons with early-onset AD scored lower on tasks measuring attention, praxis and verbal learning compared to those with late-onset AD.^17^

This heterogeneity in clinical presentations of YOD complicates the diagnosis in routine care. One challenge in diagnosing YOD lies in the diagnostic criteria for dementia, which have been based on LOD and emphasize episodic memory impairment.^18^ Consequently, patients who present with cognitive decline without memory impairment, which is thus more frequent in YOD, can have a longer time to diagnosis.^11,19^ Research has shown that the average time to diagnosis in LOD is 2.8 years, whereas in YOD this extends to 4.4 years.^20^ Other reasons for diagnostic delays include longer time before seeking medical advice, denial or hiding of early symptoms by patients, unfamiliarity of YOD among clinicians, or misdiagnosis.^12,21^ Common misdiagnoses for persons with YOD include depression or other psychiatric disorders, due to overlapping symptoms such as behavioral and personality changes caused by YOD.^22,23^

Recognizing the first symptoms of YOD in general practice (GP) is crucial for early diagnosis, as GPs in the Netherlands act as gatekeepers to more specialized services and hospital care.^24^ In practice, this means that the GP must first identify sufficient signs or concerns before patients can be referred to specialized memory clinics or other specialist services. This places primary responsibility for early recognition squarely on the GP. Timely recognition of the condition enables individuals with YOD and their families to receive adequate support to help them cope with the disease and planning for the future, particularly regarding work and family life. This improves quality of life.^12,20^ While previous studies have compared YOD to age-matched controls without dementia,^10^ no direct comparison of early symptoms between YOD and LOD has been conducted. This comparison is crucial to identify distinct symptom patterns specific to YOD, which could enhance GP awareness of YOD and facilitate more accurate and timely referrals. Therefore, the aim of this study is to investigate the early symptoms of persons with YOD in general practice up to five years prior to diagnosis and compare these symptoms to those of persons with LOD.

## Methods

A nested case-control study within the Research Network Family Medicine (RNFM) was conducted to retrospectively analyze available data on the presence of pre-diagnostic signs and symptoms in YOD and LOD to identify differences in symptoms up to five years before diagnosis. The RNFM database contains continuously collected clinical data from GP-records in the south-eastern part of the Netherlands on their patient encounters. For this study, data from January 1, 2014, to December 31, 2019, were used. All data in this study were anonymized.

### Participants

The cases in this study were individuals diagnosed with YOD between 2016 and 2019. Cases were included starting from 2016 to ensure each case had at least one full year of data available. An age cut-off of 70 years was applied to account for delayed diagnoses, as previous research has shown that the average time to diagnosis in YOD is 4.4 years.^20^ The cases were identified by an ICPC (International Classification of Primary Care) code of P70, indicating a dementia diagnosis. ICPC codes are a standard international coding system in which a diagnosis or problem description can be ticked and coded to classify reasons of healthcare encounters.^25^ In routine practice, GPs enter a P70 code when a dementia diagnosis has been established or confirmed, often following a referral to and subsequent feedback from a memory clinic or specialist. Therefore, the dementia diagnoses in this study reflect recognized clinical cases as recorded in GPs’ electronic health records.

**Figure 1.**
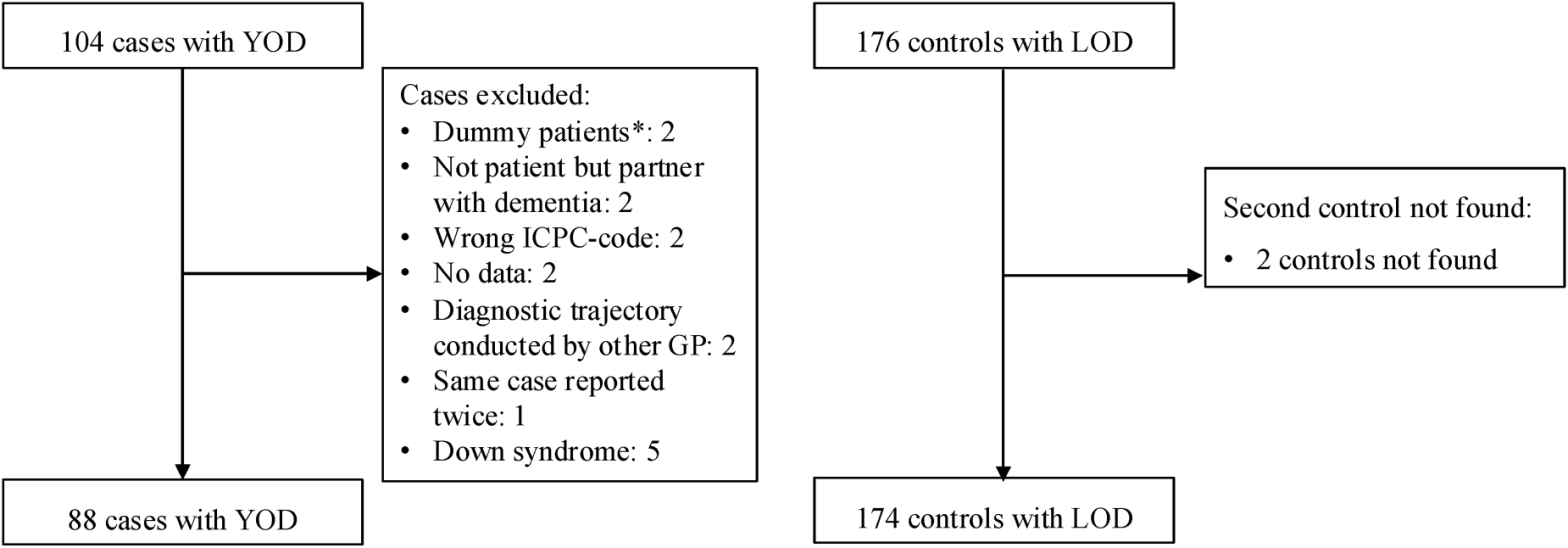
Flow-chart of in- and excluded cases and controls. *Dummy patients were patient files that did not belong to a single patient.

104 YOD cases were initially selected from the RNFM database. Of these, 16 cases were excluded due to various reasons such as insufficient information or having Down syndrome (Figure 1). Persons with Down syndrome have a higher risk of developing YOD and cannot be compared to LOD patients without Down syndrome.^26^ After exclusion, 88 YOD cases were included in this study.

For every case with YOD, two control patients with LOD were matched by sex and GP practice. The inclusion criterion for the control patients was that they needed to have received a dementia diagnosis (by means of an ICPC P70 code) after the age of 70. For two YOD cases, matching a second control was not possible. Therefore, 174 LOD controls were included in this study.

Since the number of available YOD cases was fixed at 88, this sample size was used as the starting point for assessing statistical power. To determine what effect size could be detected with this sample size, a power analysis conducted in a previous study by Hendriks et al. using the same database was used.^10^ This analysis showed that with at least 84 cases and 84 controls, an odds ratio (OR) of 2.0 could be detected. Therefore, our sample size of 88 YOD cases and 174 LOD controls is sufficient to detect this effect size (for details, see Supplemental Method 1).

### Data collection

This study used data on signs, symptoms, and diagnoses from GP appointments up to five years prior to a dementia diagnosis. The data were obtained from the RNFM database, which contains routinely collected clinical information from GP records. More specifically, the data for this study were extracted from routine clinical notes written by GPs during or immediately after consultations with patients. These notes form part of each patient's electronic health record and are collected for clinical care purposes rather than for research specifically.

GP notes may include information reported directly by patients, concerns mentioned by relatives, or signs observed by GPs during consultations. As the source of each observation could not always be distinguished, all information was extracted as documented in the GP notes, However, when relatives’ concerns about cognitive or behavioural changes were explicitly mentioned, these were coded separately under the ‘*worries from friends/family’* subcategory.

To enhance statistical power, signs and symptoms were clustered into eight categories. The categories used in this study were based on categories from the previous study by Hendriks et al. which compared first symptoms in YOD to age-matched controls without dementia to allow cross-study comparisons.^10^ The categories were 1) cognitive symptoms; 2) affective symptoms; 3) behavioral symptoms; 4) vascular symptoms; 5) gait disturbances; 6) changes in weight or appetite; 7) social indicators 8) disturbances in daily functioning.

In this study, non-clinical observations that were recorded in GP notes were also included. We used the term social indicators to denote non-clinical observations that relate to the person's social environment or the concerns of significant others. Some recorded items (e.g., worries from friends/family or fear of dementia) do not represent clinical symptoms. However, they were included to capture the psychosocial context of initial presentations in primary care and to reflect how GPs document initial concerns in routine practice. This provides a more holistic view of the pre-diagnostic phase at the General Practice.

The list of symptom categories and the signs and symptoms included in each category can be found in Supplemental Method 2. Several signs and symptoms were described in broad terms, as GPs did not always specify the nature or subtypes of these presentations. Therefore, prior to data extraction, consensus definitions were established to determine which observations would be coded under these broader symptom labels. To increase transparency, the interpretation of selected broad signs and symptoms is briefly outlined here. Confusion was recorded when it was observed by the GP, reported by caregivers, or inferred from the patient's narrative. Cognitive decline was recorded when the GP explicitly noted “cognitive decline” or when documented observations indicated a general pattern of cognitive deterioration. Language problems included any documented difficulties related to speech or language use. Similarly, sleep-related problems encompassed all recorded mentions of sleep difficulties.

Patient records were coded by two researchers (SH for YOD, CH for LOD) using the established coding categories and decision rules. Both researchers had access to each other's coded files, allowing continuous comparison and alignment during coding. Any uncertainties or ambiguous cases were discussed collaboratively and resolved through consensus to maintain consistency across the dataset.

The years of available data per person varied based on their diagnosis year, as retrospective data only went back to 2014. For instance, individuals diagnosed in 2019 could have a maximum of five years of data, while those diagnosed in 2016 had just two years.

### Data analysis

Statistical analyses were performed using IBM SPSS Statistics for Windows (version 26.0). Baseline characteristics of both groups were determined using descriptive statistics. These characteristics include male-to-female ratio, mean age, age range, whether they had a case manager in the years prior to their diagnosis, years of retrospective data available, and subtype of dementia when available. The main analyses were conducted using binary logistic regression, with group (YOD, LOD) as the independent variable and symptom presence (yes, no) as the dependent variable, yielding ORs and their 95% confidence intervals (CI). This was used to examine the differences of the symptom categories between the two groups. First, the differences in odds of symptom categories between groups were analyzed for the entire five years before diagnosis, to see if there is an overall difference between the groups per symptom category. Presence of a symptom category was defined as having at least one reported corresponding symptom over the course of the entire five years. The analyses were repeated for each individual year prior to diagnosis, to indicate more specifically at what point the differences between the groups were significant. For each year, only individuals with GP data available up to that time point prior to diagnosis were included. Persons whose GP records did not extend back to that specific year before diagnosis were excluded from the analysis for that year, rather than being classified as symptom-free. As a subanalysis, we performed the same analyses for males and females separately. Statistical significance was set at p < .05 in two-sided tests.

In the main analysis, we used an age cut-off of 70 years to distinguish between YOD and LOD. Given the diagnostic delay of about 5 years in YOD, this decision was made to ensure that no YOD cases were mistakenly included in the LOD group if the cut-off was set at 65 years. It is acknowledged that this cut-off may include persons in the YOD group who, according to the formal definition, would be classified as having LOD. To assess the robustness of our findings under a stricter threshold, we conducted a sensitivity analysis with the age cut-off at 65. In this analysis, the YOD group included only individuals diagnosed at age 65 or younger (n = 51), and the LOD group included individuals diagnosed at age 66 or older (n = 211). Due to the stricter age cut-off, it was not possible to maintain the original 1:2 matching on sex and GP practice, as no additional YOD cases were available. While it would have been possible to randomly select a matched subsample of 102 LOD individuals, to maintain the 1:2 ratio this way, this would have resulted in a considerable loss of data and statistical power. Instead, we retained all available LOD controls and included sex and GP practice as covariates to adjust for potential confounding.

### Ethical approval

The study was approved by the Medical Ethics Committee of the Faculty of Health, Medicine and Life Sciences of Maastricht University, Maastricht, the Netherlands (FHML-REC/2020/115). According to their guidelines, the study was not covered by the National Medical Research on Human Subjects Act.

## Results

Baseline characteristics of the study population are presented in Table 1. The mean age at diagnosis was 62.5 years for persons with YOD and 83.8 years for persons with LOD. Slightly more women than men were included in this study: 54.5% of those with YOD and 55.2% of those with LOD were women. None of the YOD cases had a dementia case manager before diagnosis, compared to 10.3% of LOD cases. Dementia subtypes were included if the GP had recorded them in the patient's medical file. For those with YOD, subtypes were documented for 52.3% of cases, and for those with LOD, the percentage was 32.2%. The most commonly documented subtypes in both groups were AD (YOD = 26.1%, LOD = 20.1%) and vascular dementia (YOD = 11.4%, LOD = 7.5%).

**Table 1.** Baseline characteristics of the study sample.

	Persons with YOD (*n* = 88)	Person with LOD (*n* = 174)
Age, (mean, range)	62.5 (32–69)	83.8 (74–100)
Female, (*n*, %)	48 (54.5%)	96 (55.2%)
Case manager prior to diagnosis (*n*, %)	0 (0.0%)	18 (10.3%)
Subtype of dementia (if known)*		
Alzheimer's disease (*n, %*)	23 (26.1%)	35 (20.1%)
Vascular dementia (*n, %*)	10 (11.4%)	13 (7.5%)
Mixed dementia (*n, %*)	2 (2.3%)	3 (1.7%)
Primary progressive aphasia (*n, %*)	1 (1.1%)	-
Parkinson's dementia (*n, %*)	3 (3.4%)	3 (1.7%)
Lewy body dementia (*n, %*)	1 (1.1%)	-
Frontotemporal dementia (*n, %*)	3 (3.4%)	2 (1.1%)
Secondary dementia** (*n, %*)	3 (3.4%)	-
Unknown (*n, %*)	42 (47.7%)	118 (67.8%)
Retrospective information available before diagnosis		
1 year (*n*)	88	174
2 years (*n*)	82	168
3 years (*n*)	74	160
4 years (*n*)	59	131
5 years (*n*)	36	93
6 years (*n*)	3	17

*Not all subtypes of diagnoses were documented; **Secondary dementias were due to Huntington's disease, brain tumor, multiple sclerosis.

### Difference of symptom categories between YOD and LOD cases

Odds ratios for the entire five years before diagnosis are presented in Table 2. Persons with YOD had significantly higher odds of reporting affective, behavioral, and social indicators, and significantly lower odds of reporting gait disturbances compared to persons with LOD anytime during the five years preceding diagnosis.

**Table 2.** Odds ratios with 95% confidence interval (CI) of symptom categories present anytime during the maximum available data prior to diagnosis (up to five years).

	Odds ratio (95% CI)	*p*
Cognitive symptoms	1.223 (0.936–1.597)	0.139
Affective symptoms	2.230 (1.629–3.054)	**<0**.**001**
Behavioral symptoms	1.558 (1.068–2.273)	**0**.**021**
Vascular symptoms	0.719 (0.465–1.112)	0.138
Gait disturbances	0.535 (0.379–0.754)	**<0**.**001**
Changes in weight or appetite	1.367 (0.914–2.045)	0.128
Social indicators	2.752 (2.002–3.784)	**<0**.**001**
Daily functioning disturbances	1.495 (0.966–2.313)	0.071

All cases and controls were included in this analysis, regardless of the years of data they had available.

Table 3 presents the ORs for each symptom category by year prior to diagnosis, along with the percentages of persons with YOD and LOD reporting these symptoms. It shows that persons with YOD had significantly higher odds than those with LOD of reporting affective symptoms two years and one year before diagnosis. Also, persons with YOD showed higher odds of social indicators at five, four, and one year before diagnosis. Additionally, persons with YOD had significantly lower odds of reporting gait disturbances at four and two years before diagnosis.

**Table 3.** Odds ratios (OR) with 95% confidence interval (CI) of the symptom categories over the maximum available data before diagnosis and number of persons with YOD and persons with LOD reporting symptoms over the maximum available data before diagnosis.

Symptom category	Odds ratio (95% CI)	*p*	Persons with YOD	Persons with LOD
Cognitive symptoms				
1 year before diagnosis	1.014 (0.513–2.003)	0.968	73/88 (83.0%)	144/174 (82.8%)
2 years before diagnosis	1.626 (0.923–2.863)	0.092	30/82 (36.6%)	44/168 (26.2%)
3 years before diagnosis	0.820 (0.403–1.669)	0.585	13/74 (17.6%)	33/160 (20.6%)
4 years before diagnosis	1.369 (0.585–3.201)	0.469	10/59 (16.9%)	17/131 (13.0%)
5 years before diagnosis	0.723 (0.143–3.655)	0.695	2/36 (5.6%)	7/93 (7.5%)
Affective symptoms				
1 year before diagnosis	2.363 (1.352–4.130)	**0.003**	35/88 (39.8%)	38/174 (21.8%)
2 years before diagnosis	2.425 (1.303–4.512)	**0.005**	26/82 (31.7%)	27/168 (16.1%)
3 years before diagnosis	1.633 (0.774–3.447)	0.198	14/74 (18.9%)	20/160 (12.5%)
4 years before diagnosis	2.318 (0.986–5.446)	0.054	12/59 (20.3%)	13/131 (9.9%)
5 years before diagnosis	1.867 (0.613–5.686)	0.272	6/36 (16.7%)	9/93 (9.7%)
Behavioral symptoms				
1 year before diagnosis	1.328 (0.761–2.317)	0.317	29/88 (33.0%)	47/174 (27.0%)
2 years before diagnosis	2.072 (0.925–4.643)	0.077	13/82 (15.9%)	14/168 (8.3%)
3 years before diagnosis	1.818 (0.687–4.814)	0.229	8/74 (10.8%)	10/160 (6.3%)
4 years before diagnosis	1.114 (0.198–6.258)	0.902	2/59 (3.4%)	4/131 (3.1%)
5 years before diagnosis	N/A*		0/36 (0.0%)	2/93 (2.2%)
Vascular symptoms				
1 year before diagnosis	0.877 (0.382–2.016)	0.758	9/88 (10.2%)	20/174 (11.5%)
2 years before diagnosis	0.560 (0.231–1.360)	0.200	7/82 (8.5%)	24/168 (14.3%)
3 years before diagnosis	0.625 (0.268–1.455)	0.275	8/74 (10.8%)	26/160 (16.3%)
4 years before diagnosis	1.120 (0.365–3.435)	0.842	5/59 (8.5%)	10/131 (7.6%)
5 years before diagnosis	0.304 (0.037–2.518)	0.269	1/36 (2.8%)	8/93 (8.6%)
Gait disturbances				
1 year before diagnosis	0.619 (0.355–1.077)	0.089	25/88 (28.4%)	68/174 (39.1%)
2 years before diagnosis	0.430 (0.203–0.910)	**0.027**	10/82 (12.2%)	41/168 (24.4%)
3 years before diagnosis	0.533 (0.241–1.180)	0.121	9/74 (12.2%)	33/160 (20.6%)
4 years before diagnosis	0.326 (0.119–0.889)	**0.029**	5/59 (8.5%)	29/131 (22.1%)
5 years before diagnosis	0.237 (0.029–1.923)	0.178	1/36 (2.8%)	10/93 (10.8%)
Changes in weight or appetite				
1 year before diagnosis	1.119 (0.561–2.232)	0.750	15/88 (17.0%)	27/174 (15.5%)
2 years before diagnosis	1.200 (0.559–2.577)	0.640	12/82 (14.6%)	21/168 (12.5%)
3 years before diagnosis	2.077 (0.806–5.351)	0.130	9/74 (12.2%)	10/160 (6.3%)
4 years before diagnosis	1.424 (0.445–4.551)	0.551	5/59 (8.5%)	8/131 (6.1%)
5 years before diagnosis	1.035 (0.192–5.593)	0.968	2/36 (5.6%)	5/93 (5.4%)
Social indicators				
1 year before diagnosis	3.995 (2.326–6.864)	**<0.001**	56/88 (63.6%)	53/174 (30.5%)
2 years before diagnosis	1.901 (0.920–3.927)	0.083	16/82 (19.5%)	19/168 (11.3%)
3 years before diagnosis	2.018 (0.883–4.612)	0.096	12/74 (16.2%)	14/160 (8.8%)
4 years before diagnosis	3.750 (1.269–11.085)	**0.017**	9/59 (15.3%)	6/131 (4.6%)
5 years before diagnosis	4.839 (1.092–21.436)	**0.038**	5/36 (13.9%)	3/93 (3.2%)
Daily functioning disturbances				
1 year before diagnosis	1.501 (0.838–2.687)	0.172	26/88 (29.5%)	38/174 (21.8%)
2 years before diagnosis	1.910 (0.709–5.148)	0.201	8/82 (9.8%)	9/168 (5.4%)
3 years before diagnosis	2.194 (0.303–15.889)	0.437	2/74 (2.7%)	2/160 (1.3%)
4 years before diagnosis	N/A*	0.690	0/59 (0.0%)	3/131 (2.3%)
5 years before diagnosis	0.636 (0.069–5.888)		1/36 (2.8%)	4/93 (4.3%)

*Not applicable, in one or both of the groups zero persons experienced symptoms from this category in this year, therefore no OR has been determined.

Analyses were repeated separately for males and females as a subanalysis. Here, males with YOD had significantly higher odds than those with LOD of reporting affective symptoms at five and one year before diagnosis, behavioral symptoms from two years before diagnosis, and social indicators at three and one year before diagnosis (Supplemental Result 2). For females, those with YOD have significantly higher odds than those with LOD of reporting cognitive symptoms at two years before diagnosis, affective symptoms from two years before diagnosis, and social indicators at four and one year before diagnosis (Supplemental Result 3).

### Frequency of reported symptoms

The frequency of signs and symptoms from GP records was counted within each category and visualized in bar charts, but no statistical tests were performed due to small frequencies (Supplemental Result 1). Visually, they provide insight into symptom patterns in YOD (n = 88) and LOD (n = 174) by highlighting which signs and symptoms were most commonly recorded within each category for both groups. In the cognitive category, YOD cases mostly reported forgetfulness, while LOD cases reported cognitive decline, forgetfulness, and confusion. Language problems were reported in YOD cases, and despite the smaller group size, the number of reports was higher than in LOD. In terms of affective symptoms, both groups commonly reported depressed mood and anxiety, but YOD cases more often mentioned loss of initiative and suicidal ideation; these findings are presented cautiously, as such signals are not specific to YOD and may reflect other underlying conditions requiring clinical attention. In behavioral symptoms, changes in character were most frequently reported in both groups. Chest pain was the most reported vascular symptom, especially in LOD. Falls were the most common gait disturbance. In YOD cases, weight changes were commonly reported, while in LOD, weight changes and loss of appetite were reported at comparable frequencies. YOD cases showed a wider variety of social indicators. Both groups had similar reports of worries from friends/family and relationship problems, while fear of dementia was mentioned more often in YOD. In daily functioning, both groups most commonly reported a decline in functionality.

### Sensitivity analysis

The same analyses were conducted using the stricter age cut-off of 65-years for defining YOD and LOD. These results can be found in Supplemental Result 4. Social indicators remained significantly more prevalent in persons with YOD at five, four and one year prior to diagnosis, and additionally reached significance at three years prior to diagnosis. In contrast, affective symptoms at two and one year prior to diagnosis were no longer statistically significant. Cognitive symptoms became significantly more common two years prior to diagnosis, and vascular symptoms at three years prior to diagnosis. Gait disturbances remained significantly more common in LOD at four years prior to diagnosis but not at two years prior to diagnosis.

## Discussion

This study compared onset of early signs and symptoms of persons with YOD and LOD as presented to the GP up to five years (y) before the diagnosis of dementia was made. We note that the broad symptom categories reflect how signs and concerns are recorded in routine GP notes and may differ in nature from those in medical specialists’ records. Significant differences in symptom category presence were identified. Social indicators (5y, 4y, 1y) and affective symptoms (2y, 1y) were more common in YOD defined by age at diagnosis < 70 years, while gait disturbances (4y, 2y) were less common. Subanalyses revealed that men with YOD more often exhibited affective (5y, 1y), behavioral (2y, 1y), and social indicators (3y, 1y) than men with LOD. Women with YOD also showed higher prevalence of affective (2y, 1y) and social indicators (4y, 1y), but additionally experienced more cognitive (2y) symptoms than women with LOD. These sex-specific findings should be interpreted with caution, given the smaller subgroup sizes and the exploratory nature of these analyses.

One of the most profound differences in signs and symptoms between persons with YOD and LOD in this study was within the social indicator category, where persons with YOD more often reported social indicators five years, four years, and one year before diagnosis. Earlier studies support this by showing financial problems, relationship difficulties and early retirement as common social indicators in persons with diagnosed YOD (see our Supplemental Result 1).^22^ In the study by Draper & Withall, these symptoms were suggested to be consequences after a YOD diagnosis.^22^ However, our results suggest that such social indicators are not limited to the post-diagnostic phase but can already occur in the years leading up to diagnosis. In both persons with YOD and persons with LOD, worries from friends and family were the most frequently reported social indicator (Supplemental Result 1). Persons with YOD also showed a wider range of social indicators than persons with LOD, including job loss and work problems, which seems directly linked to their active life phase at a younger age.^22^ Another notable finding is that for persons with YOD, social indicators substantially increased from 19.5% two years before diagnosis, to 63.6% one year before diagnosis. For persons with LOD this increase was far less steep. This sharp rise in social indicators in YOD may be related to the unexpected nature of YOD, but also to the many social roles that individuals may still be fulfilling at this relatively young age.^22^ It should be noted that some of the differences in social indicators between YOD and LOD may be influenced by life-stage factors, such as employment and family responsibilities, rather than being solely attributable to the underlying dementia. The sensitivity analysis using an age cut-off of 65 years showed that social indicators remained significantly more prevalent in persons with YOD at five, four, and one year prior to diagnosis, and additionally reached significance at three years prior to diagnosis. This reinforces the robustness of this finding and highlights social indicators as a potentially important early indicator of YOD, distinguishing it from LOD. The consistent presence of social indicators several years before diagnosis suggests they could serve as a valuable clinical cue for earlier recognition of YOD.

Next, affective symptoms were found to be more common in persons with YOD than LOD at two years and one year before diagnosis. In this sample, the most frequently reported affective symptoms by both groups were anxiety and depressive mood (Supplemental Result 1). While statistical significance was not tested, persons with YOD reported these symptoms more frequently in absolute numbers, despite their smaller group size. These results are consistent with previous studies showing more affective symptoms in YOD presenting to specialized memory clinics for diagnosis, but also show that such symptoms already build up in the years prior to diagnosis.^27,28^ In addition, our prior study in the same GP database, which compared pre-diagnostic signs and symptoms in persons with YOD and healthy controls, showed that affective symptoms were significantly more common in persons with YOD, presenting as early as four years before diagnosis.^10^ In contrast, in LOD, a study by Ramakers et al. in the same GP database found that affective symptoms became more common only one year before diagnosis compared with healthy controls.^29^ This suggests that affective symptoms can be an important early indicator of YOD. However, our sensitivity analysis using an age cut-off at 65 years showed that affective symptoms were no longer statistically significant at two and one year before diagnosis. This may suggest that, although common, affective symptoms may be less effective in distinguishing the pre-diagnostic profile of YOD from LOD than initially suggested. Still, it is important to note that the reduced statistical power due to the smaller YOD sample size in the sensitivity analysis could have contributed to this loss of significance. The direction of the association remained consistent, with odds ratios still above 1 at both time points (OR = 1.776 at two years and OR = 1.525 at one year before diagnosis in the sensitivity analysis, compared to ORs of 2.425 and 2.363 respectively in the main analysis). At the same time, the decrease in the odds ratios, particularly at one year prior to diagnosis, may indicate that the strength of the association is weaker in this younger age group up to age 65. These results underline the need for further research into the exact role of affective symptoms in the early identification of YOD. The presence of these symptoms, however, may also complicate the distinction between YOD and common anxiety or mood disorders, which are more frequent in younger adults. This overlap could contribute to diagnostic delays, further emphasizing the importance of distinguishing between YOD and age-matched individuals with mood or anxiety disorders without dementia. Importantly, clinicians must also be cautious to not misattribute common affective symptoms in the general population to YOD, as this could lead to unnecessary anxiety, stigma, or overdiagnosis. Striking the right balance between early detection and avoiding false positives remains a key clinical and ethical challenge. Additionally, because YOD occurs at a life stage where individuals are actively engaged in work, family life, and social roles, the unexpected onset of cognitive decline, along with the (self-)stigma it may bring, can lead to profound disruptions in identity, career, and relationships. This may contribute to increased affective symptoms, as individuals struggle to come to terms with their diagnosis and its impact on their future. In contrast, persons with LOD are typically beyond working age and may experience cognitive decline as a more expected part of aging, which could explain why affective symptoms emerge closer to diagnosis, after memory problems have aggravated, rather than in the years preceding it. Another possible explanation for the higher occurrence of affective symptoms in YOD may lie in the distribution of dementia subtypes. In particular, the behavioral variant of FTD is characterized by prominent behavioral and personality changes, including symptoms such as loss of interest, irritability, and loss of initiative, which overlap with several symptoms included in our affective symptom category.^30^ While only a small subgroup in our sample had a documented FTD diagnosis, many cases had an unknown dementia subtype. This means that the true number of persons with FTD in our study could be higher, as FTD is the second most common cause of YOD.^31^ These results should be interpreted with caution, because of the many unknown dementia subtypes in the sample, making it difficult to determine the exact contribution of FTD to the observed patterns. An important focal point for future research should be to further investigate the differences between pre-diagnostic YOD and age-matched individuals with anxiety and mood disorders who do not have dementia. This will be important for improving differentiation between these groups, ultimately reducing the amount of pre-diagnostic misdiagnoses of anxiety or mood disorders in persons with YOD. Based on the findings of the current study, the clinical implications for GPs remain somewhat limited, given that this was a retrospective study. This highlights the need for more prospective knowledge to guide earlier and more accurate recognition of YOD in clinical practice, which is crucial to ensure persons get adequate help and support.

Our results further show that gait disturbances are less common in persons with YOD compared to LOD. Previous studies support this finding, showing that gait disturbances are equally common in healthy controls and persons with YOD in the pre-diagnostic phase,^10^ whereas they are more common in individuals with LOD compared to age-matched controls without dementia at five, three, and one year prior to diagnosis.^29^ This difference may be explained by higher levels of physical frailty in persons with LOD compared to those with YOD.^32^ Another possible explanation relates to differences in dementia subtype distribution across age groups. Motor symptoms such as gait disturbances and a tendency to fall are known symptoms of vascular dementia, particularly subcortical ischaemic vascular dementia, which occurs more frequently at older ages.^33^ Although the proportion of vascular dementia among cases with a known subtype was higher in YOD than in LOD in our sample, subtype information was unavailable for a large proportion of individuals (67.8% in LOD). Given that vascular dementia is the second most common dementia subtype in LOD, it is plausible that additional vascular dementia cases are present among those with unknown subtype in the LOD group.^33^ Consequently, differences in subtype distribution may partly contribute to the higher frequency of gait disturbances observed in LOD. However, this explanation should be interpreted with caution given the substantial proportion of missing subtype data.

Behavioral symptoms were again more common in persons with YOD than LOD, though analyses for each year separately showed no significant differences, questioning its use for differentiation between YOD and LOD. Previous research found that behavioral symptoms were more frequently reported up to two years prior to diagnosis in persons with YOD compared to age-matched controls without dementia.^10^ Similarly, a separate study found that such symptoms were also more common one year before diagnosis in persons with LOD compared to their age-matched controls without dementia.^29^ These findings suggest that while behavioral symptoms may signal an emerging dementia syndrome in both groups, they may not be specific enough to aid early recognition of YOD in clinical practice. In the subanalyses, we found that men with YOD in particular showed more behavioral symptoms than men with LOD. One possible explanation is that the behavioral variant of FTD, which is more common in men, usually includes symptoms such as personality changes, emotion regulation issues, agitation, irritability, and aggression, similar to those identified in this study (Supplemental Result 1).^34,35^ While only a small subgroup in our sample is known to have FTD, many cases did not have a dementia subtype diagnosis documented, meaning that the true number of persons with FTD could be higher, as FTD is the second most common cause of YOD.^31^ These results should be interpreted cautiously given the exploratory nature of the sex-stratified analyses and the relatively small subgroup sizes. Future studies with larger samples are needed to validate these sex-specific findings.

The absence of significant differences for cognitive symptoms between persons with YOD and LOD was unexpected. Prior research suggests persons with YOD typically present with fewer cognitive symptoms, especially in the early stages.^12,19^ This result may be due to several factors. Firstly, GPs’ heuristics may influence the way in which these symptoms are interpreted and recorded. In younger patients with subtle cognitive changes, there may be also work-related stress, mood disorders, or burnout, whereas similar complaints in older patients are more likely to be regarded as early-stage dementia. This emphasizes that equivalent documentation does not necessarily imply identical clinical recognition. It is possible that persons with YOD experience cognitive symptoms as frequently as persons with LOD, but that these symptoms are often attributed to other psychosocial stressors rather than dementia, delaying the consideration of a dementia diagnosis. In contrast, when older individuals exhibit cognitive symptoms, dementia is considered earlier, and cognitive decline is promptly explained by this. Secondly, persons with LOD, on average, experience a 2.8-year time to diagnosis and often present cognitive symptoms early on.^9,20^ In contrast, persons with YOD typically experience a 4.4-year time to diagnosis, with cognitive symptoms often emerging later.^12,20^ This could explain why cognitive symptoms appear around the same time in both groups when looking retrospectively at the years prior to diagnosis, and why symptoms increase as diagnosis approaches, as persons with YOD are more likely to be further in their disease process at the time of comparison. This is indeed a point to consider for all the analyses. However, the point at which the formal diagnosis is made in the pre-diagnostic phase is also variable depending on the case. It is possible that the GP would postpone the formal diagnosis to avoid causing unnecessary distress for their patient. This applies to both YOD and LOD. However, since individuals with YOD are often still working and managing social roles, an earlier diagnosis may be more desirable to allow for better planning and adjustments in daily life. In contrast, for those with LOD, postponing a formal diagnosis might be preferable to avoid unnecessary stress, as the diagnosis may have less immediate impact on their daily responsibilities. Moreover, although this study used all of GPs’ notes from their records, it is possible that GPs may not have documented cognitive symptoms in older patients anyway because they expect these symptoms more often in that age group. On the other hand, when a younger person with YOD experiences cognitive decline, the GP may consider this more noteworthy and therefore document it in the patient record. Finally, the cognitive symptom category used in this study was quite broad. The lack of a significant difference could be due to the inclusion of symptoms like confusion and forgetfulness, which may be more common in LOD than YOD, alongside symptoms like language and orientation problems, which may be more common in YOD.^9,36^ Combining these different types of cognitive symptoms into a single category may have concealed potential differences between persons with YOD and LOD.

No significant differences were found for presence of vascular symptoms, changes in weight or appetite, and daily functioning. In line with these findings, our previous study comparing persons with YOD with age-matched controls without dementia found no significant differences in weight or changes in appetite.^10^ Similarly, Ramakers et al. compared persons with preclinical LOD to age-matched controls without dementia, and found weight or appetite changes were more common in persons with LOD only one year prior to diagnosis.^29^ These findings suggest that weight or appetite changes are not strong early indicators for YOD or LOD in primary care settings. Furthermore, the lack of significant differences in vascular symptoms and daily functioning in our study further suggests that these factors are not useful for differentiating early symptoms of YOD or LOD, which is essential for early diagnosis in primary care.

In addition, our findings may highlight the potential benefit of more collaborative models of dementia care. In countries such as Canada, Primary Care Collaborative Memory Clinics (PCCMCs) integrate specialist expertise directly into primary care. This allows family physicians to consult with geriatricians and other specialists early in the diagnostic trajectory.^37^ Such models reduce the diagnostic burden on primary care and facilitate earlier recognition in complex or atypical cases, including YOD.^37^ Although the Dutch healthcare system operates on a strict GP gatekeeping model, incorporating certain elements of collaborative memory clinic structures could promote early detection. These elements include earlier specialist input, shared assessment pathways, and enhanced support for GPs. Future research may therefore explore whether adapting aspects of these models could improve the feasibility and accuracy of diagnoses in the Dutch context.

An interesting secondary finding was that one in ten persons with LOD had a dementia case manager prior to diagnosis, compared to none in the YOD group, contributing to the impression that GPs did not consider a diagnosis of dementia in the latter at earlier stages. In the Netherlands, case managers are typically the first point of contact for families and assist with planning and disease management.^38^ Dementia is more readily considered in older adults, prompting earlier involvement of case managers even before a formal diagnosis, i.e., in the stage of mild cognitive impairment. In contrast, YOD is often misattributed to stress, psychiatric conditions, or other non-neurodegenerative causes, which may delay access to specialized dementia care and support services. Additionally, case management services are often geared toward older adults, and younger individuals with cognitive symptoms may not yet be referred to these services by GPs. This highlights a gap in pre-diagnostic support for persons with YOD and their families, emphasizing the need for increased awareness and earlier referral pathways in primary care.

Finally, we also observed that dementia subtypes were more frequently recorded in GP records for persons with YOD than LOD. Additionally, a larger variety of less common dementia subtypes were documented in persons with YOD. This is in line with prior research showing that YOD is caused by a larger range of subtypes, each presenting with a diverse set of early symptoms.^11^ This emphasizes the importance of being familiar with the early symptoms of YOD.

A strength of this study is that it included data from multiple GP practices in the south-eastern part of the Netherlands. This ensures that the findings are not limited to a single patient group, making the study sample representative of individuals with YOD and LOD in the general population. In addition, the use of GP notes minimized recall bias, as notes were taken immediately after consultation.

Limitations of this study include the fact that, for reasons of efficiency, patient files were reviewed by two different researchers, one for YOD (SH) and one for LOD (CH), since the former was used in a previous study.^10^ While this allowed for cross-study comparisons using a standard mode of operation and despite prior inter-rater agreements on interpretation of data in the records, inconsistencies may have led to interpretation bias. To minimize this risk, cases of uncertainty were discussed collaboratively to ensure consistent decision-making between researchers. The study's statistical power may have been too low, as the expected OR of ≥2.0 was not met in most analyses, meaning that some meaningful differences in symptom presence may not have been detected in this study (type II error). Future research should aim to include larger sample sizes to improve the ability to detect meaningful effects. This would help ensure that relevant differences are not missed, while also resulting in larger subgroups for sensitivity analyses and thereby increasing the reliability of such analyses. Not all individuals had GP data available for the full five years prior to diagnosis, meaning that the number of participants included in the year-specific analyses varied across time points. Consequently, differences in symptom prevalence between groups across the years prior to diagnosis may partly reflect differences in opportunities for documentation in GP records rather than true differences in symptom occurrence. Age differences and varying comorbidity profiles between YOD and LOD participants may have also influenced how certain symptoms such as gait disturbances or falls were recorded in GP notes, and should be considered when interpreting these results. In the present study, the etiology of dementia was unknown for a substantial proportion of both YOD and LOD cases. Whilst the analysis of a homogeneous sample may offer more targeted insights, real-world primary care databases such as ours frequently exhibit inconsistencies with regard to the information available on the etiology of dementia. Notwithstanding this limitation, our dataset provides a unique opportunity to examine the presentation of early symptoms in the context of general practice, i.e., the context in which initial recognition occurs. Consequently, it was not feasible to restrict analyses to specific etiological subtypes, such as Alzheimer's disease or vascular dementia, without substantially reducing sample size and representativeness. It is recommended that future studies link GP data with specialist diagnostic information. The rationale for this is that it would allow researchers to focus on more homogeneous patient groups. This, in turn, would provide the opportunity to investigate differences in symptom presentation across dementia subtypes. Furthermore, the utilization of larger, well-characterized samples has the potential to facilitate additional exploration of potential sex differences and other factors.

Uncertainty about the timing of diagnoses could also have affected the retrospective data and symptom categorization, as not all GPs documented this information in detail. In addition, GP notes in the records are mainly written as memory aids and therefore may not include all symptoms or be consistently detailed across different GPs. This is also reflected in the reporting of dementia subtypes, which were recorded in only 52.3% of cases of YOD and 32.2% of cases of LOD, suggesting that certain details of the diagnostic process are not always documented in GP records. Finally, heterogeneity in notation styles across GPs may have led to some degree of symptom misclassification, for example if GPs did not note whether symptoms were reported by the patient themselves, noticed by the GP or mentioned by friends or family. As these sources may differ in their perspective and in the stage of disease at which symptoms become apparent, this lack of clarity could have reduced the precision and comparability of symptom categorization across cases. To accommodate this heterogeneity in notation styles more generally and to maintain consistency in coding, we grouped related observations into broader symptom categories. While this approach allowed for cross-study comparisons and more robust analyses, it may have obscured more subtle differences between individual symptoms. In this study, YOD was defined as dementia diagnosed before age 70 to account for potential diagnostic delay. This choice is clinically relevant for studying symptom documentation and early risk markers in primary care. We acknowledge that the YOD vs LOD dichotomy is somewhat arbitrary and that a single age cut-off may oversimplify age-related variation in symptom presentation. While an age-continuum approach or narrower age bands could be considered, this would reduce clinical interpretability and go beyond the scope of the current study. Future research could therefore explore the potential of an age-continuum approach to better understand the onset and progression of dementia, which may help refine early detection strategies and clarify age-related differences in symptom presentation.

In conclusion, this study found that persons with YOD exhibit different symptoms at the GP than those with LOD up to five years prior to diagnosis. In particular, social indicators are more prevalent in YOD. These findings highlight patterns in presentation in primary care that may serve as potential risk markers, while acknowledging that many of these features may also occur in other common conditions or daily stress. In the Netherlands, everyone is registered with a GP, who is often the first step in seeking medical care. Consequently, GPs play a crucial role in the timely recognition of symptoms and appropriate referral to specialist care. They typically refer patients with suspected YOD to specialized memory clinics or neurology departments in hospitals. Delays most often occur during the initial “pre-diagnostic” phase, when symptoms are mistakenly attributed to work-related burnout, depression, or relational stress, and further bottlenecks arise within the clinical pathway due to the complexity of diagnosing atypical presentations. Although healthcare systems vary internationally and patients in some settings may have more direct access to specialist care, awareness of these early patterns of presentation may also support clinicians in other healthcare settings. Together, these findings contribute to a better understanding of how YOD may present in primary care and provide a foundation for future research aimed at improving awareness of potential early warning patterns.

## Supplemental Material

sj-docx-1-alz-10.1177_13872877261451197 - Supplemental material for Comparison of pre-diagnostic signs and symptoms of young-onset dementia and late-onset dementia in the general practice Supplemental material, sj-docx-1-alz-10.1177_13872877261451197 for Comparison of pre-diagnostic signs and symptoms of young-onset dementia and late-onset dementia in the general practice by Caitlin Hibbs, Stevie Hendriks, Jean Muris, Ralph Leijenaar, Dorothee Horstkötter, Sebastian Köhler and Marjolein De Vugt in Journal of Alzheimer's Disease
