# Supplementary material for: Comparison of pre-diagnostic signs and symptoms of young-onset dementia and late-onset dementia in the general practice
Source: J Alzheimers Dis. 2026 May 27;112(1):513–24. doi: 10.1177/13872877261451197 (PMC13291403; doi:10.1177/13872877261451197)
Supplement: sj-docx-1-alz-10.1177_13872877261451197 - Supplemental material for Comparison of pre-diagnostic signs and symptoms of young-onset dementia and late-onset dementia in the general practice [file sj-docx-1-alz-10.1177_13872877261451197.docx]

**Supplemental Material**

**Comparison of pre-diagnostic signs and symptoms of young-onset dementia and late-onset dementia in the general practice**

**Supplemental Method 1. Power analysis/sample size calculation**

Using G-Power, a sample size calculation was performed a priori to obtain the sample size needed for this study. For this, different scenarios were assessed. In all scenarios alfa is set on 0.05, and the power was set at 0.8. Also, a normal distribution was assumed.

|  | Expected minimal OR | Proportion of patients with symptoms | Sample size needed in each group  (x2 for controls) |
| --- | --- | --- | --- |
| Scenario 1 | 2.0 | 0.3 | 92 |
| Scenario 2 | 2.0 | 0.6 | 84 |
| Scenario 3 | 1.5 | 0.3 | 242 |
| Scenario 4 | 1.5 | 0.6 | 215 |

Based on previous research, ORs can range significantly depending on the type of symptoms studied. It is difficult to calculate the needed sample size a priori. Sample sizes for several scenarios were therefore calculated. Scenario 2 was deemed most probable since previous research in elderly people with dementia found very high ORs, with increasing proportion of patients with symptoms over the 5 years before diagnosis.

**Supplemental Method 2. Categories of signs and symptoms**

A: cognitive symptoms

Forgetfulness, confusion (either mentioned by caregivers, noticed by the GP, or categorized based on the patient’s story), cognitive decline, orientation problems, language problems, problems with logical thinking

B: affective symptoms

Fatigue, irritability, anxiety, sleep related problems, depressive mood, being upset, loss of initiative, loss of interest, crying, complaining, hyperventilation, mood changes, suicidal ideation

C: behavioral symptoms

Restlessness, delusions/hallucinations, aggression/agitation, changes in character, suspicion

D: vascular symptoms

Chest pains, loss of speech, temporary paralysis, continuous paralysis, loss of strength, thick tongue

E: gait disturbances

Falls, problems with walking

F: changes in weight or appetite

Loss of appetite, weight changes

G: social indicators

*(involves everything about interaction with others)*

Worries from friends/family (about the cognitive changes they experience in their loved ones), job loss, work problems, relationship problems, financial problems, fear of dementia

H: daily functioning symptoms

*(involves everything linked to functionality of the person at home)*

Social isolation, neglect (in selfcare, household etc.), decline in functionality (difficulties with daily routine)

**Supplemental Result 1. Frequency of reported signs and symptoms**


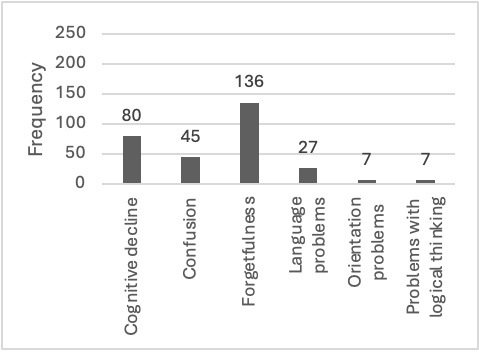


Category A: cognitive symptoms, for persons with YOD


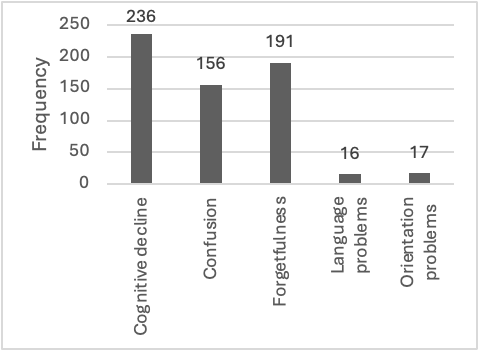


Category A: cognitive symptoms, for persons with LOD


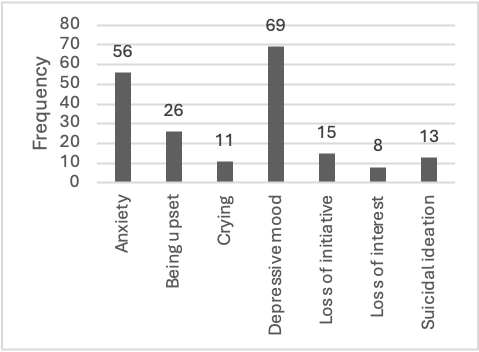


Category B: affective symptoms, for persons with YOD


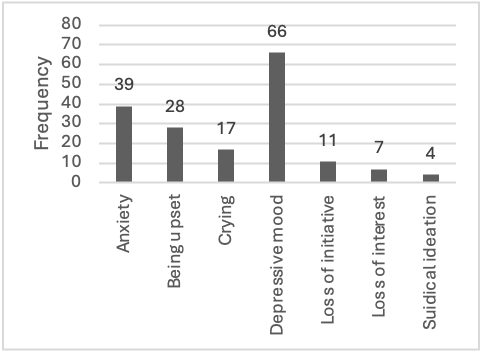


Category B: affective symptoms, for persons with LOD


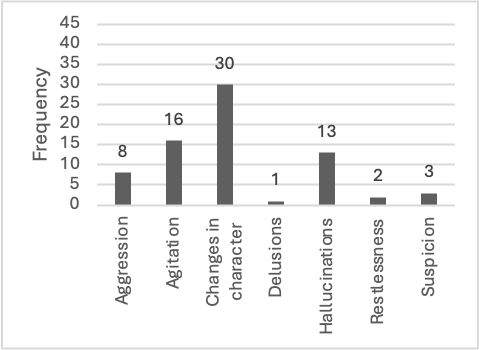


Category C: behavioural symptoms, for persons with YOD


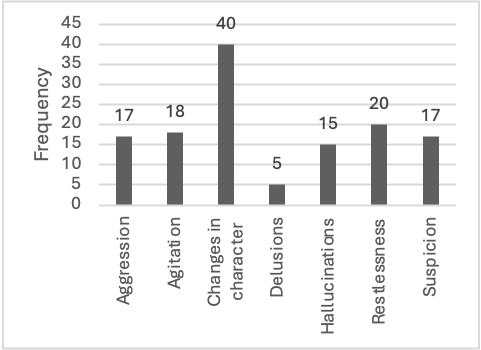


Category C: behavioural symptoms, for persons with LOD


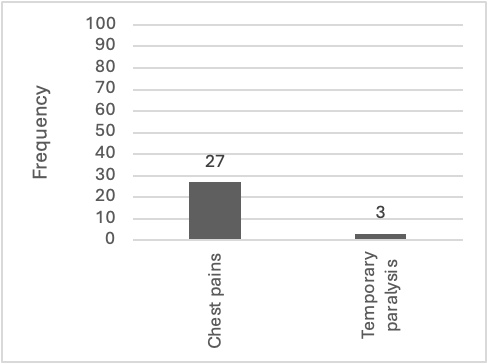


Category D: vascular symptoms, for persons with YOD


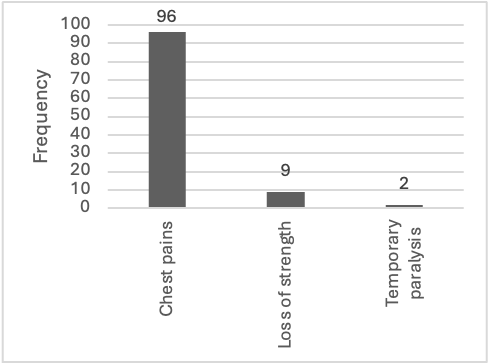


Category D: vascular symptoms, for persons with LOD


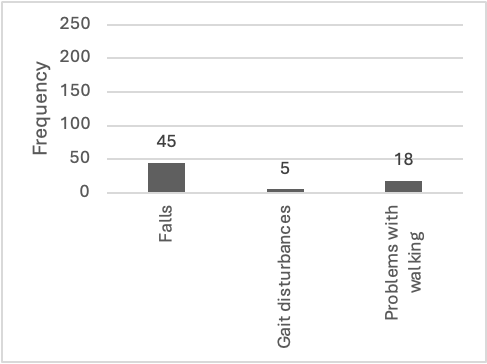


Category E: gait disturbances, for persons with YOD


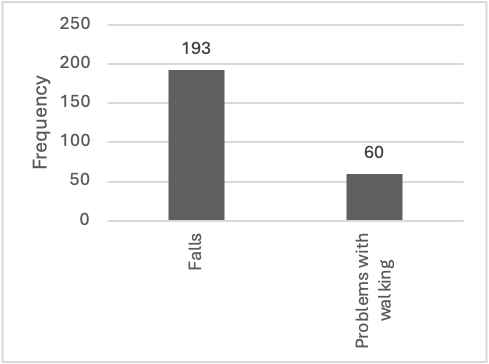


Category E: gait disturbances, for persons with LOD


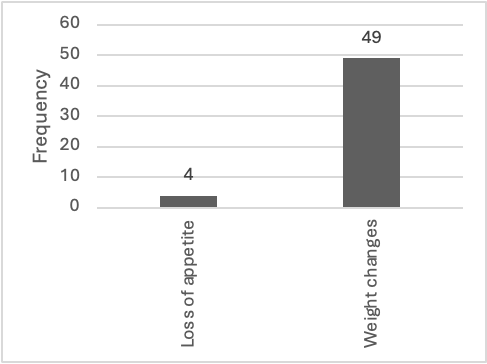


Category F: changes in weight or appetite, for persons with YOD


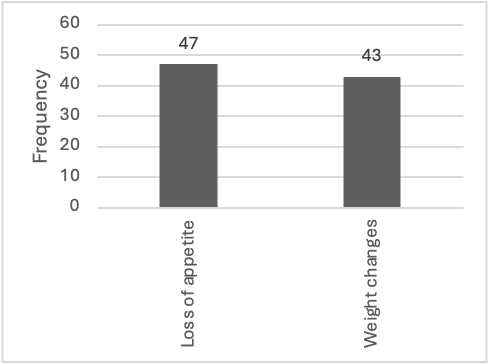


Category F: changes in weight or appetite, for persons with LOD


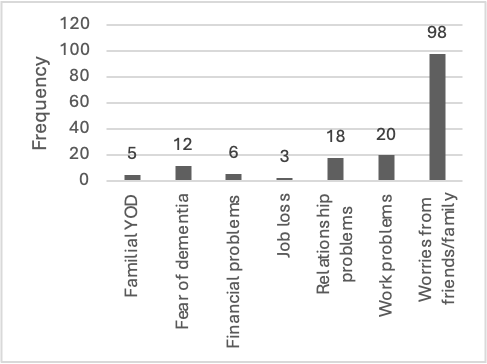


Category G: social indicators, for persons with YOD


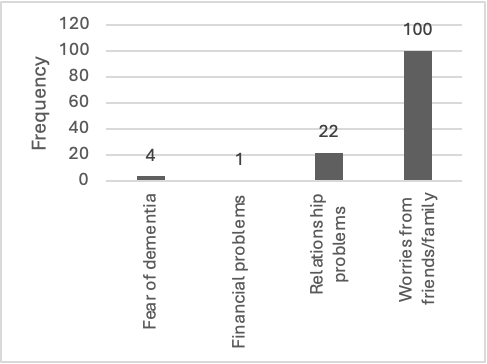


Category G: social symptoms, for persons with LOD


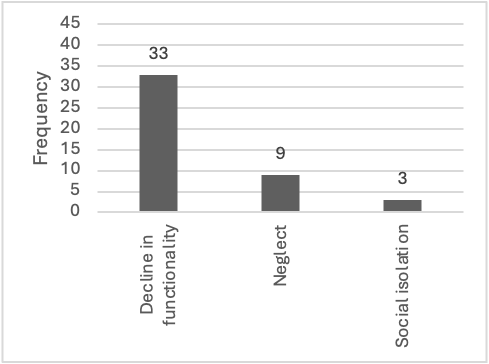


Category H: daily functioning symptoms, for persons with YOD


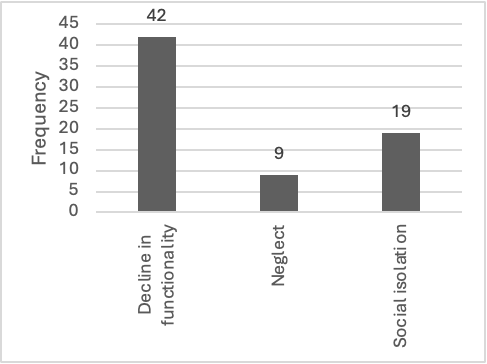


Category H: daily functioning symptoms, for persons with LOD

**Figure 2.** Frequency of signs and symptoms reported by persons with YOD and persons with LOD within the symptom categories.

*Category G: social indicators, for persons with LOD*


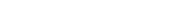


**Supplemental Result 2. Symptom ORs by year prior to diagnosis for males**

| **Supplemental Table 1.** Odds ratios (OR) with 95% confidence interval (CI) of the symptom categories over the maximum available data before diagnosis and number of males with YOD and males with LOD reporting symptoms over the maximum available data before diagnosis | | | | | |
| --- | --- | --- | --- | --- | --- |
| Symptom category | Odds ratio (95% CI) | *p* | Males with YOD | Males with LOD |  |
| Cognitive symptoms  1 year before diagnosis  2 years before diagnosis  3 years before diagnosis  4 years before diagnosis  5 years before diagnosis | 2.700 (0.847-8.609)  1.075 (0.477-2.425)  0.800 (0.279-2.296)  0.947 (0.264-3.398)  0.447 (0.047-4.296) | 0.093  0.861  0.678  0.933  0.486 | 36/40 (90.0%)  14/39 (35.9%)  6/36 (16.7%)  4/27 (14.8%)  1/20 (5.0%) | 60/78 (76.9%)  25/73 (34.2%)  14/70 (20.0%)  9/58 (15.5%)  4/38 (10.5%) |  |
| Affective symptoms  1 year before diagnosis  2 years before diagnosis  3 years before diagnosis  4 years before diagnosis  5 years before diagnosis | 2.520 (1.074-5.911)  1.873 (0.765-4.586)  1.093 (0.337-3.542)  3.029 (0.834-11.001)  12.333 (1.327-114.615) | **0.034**  0.170  0.882  0.092  **0.027** | 15/40 (37.5%)  12/39 (30.8%)  5/36 (13.9%)  6/27 (22.2%)  5/20 (25.0%) | 15/78 (19.2%)  14/73 (19.2%)  9/70 (12.9%)  5/58 (8.6%)  1/38 (2.6%) |  |
| Behavioral symptoms  1 year before diagnosis  2 years before diagnosis  3 years before diagnosis  4 years before diagnosis  5 years before diagnosis | 2.221 (0.999-4.937)  3.773 (1.030-13.819)  2.133 (0.635-7.164)  2.192 (0.132-36.427)  N/A* | **0.050**  **0.045**  0.220  0.584 | 18/40 (45.0%)  7/39 (17.9%)  6/36 (16.7%)  1/27 (3.7%)  0/20 (0.0%) | 21/78 (26.9%)  4/73 (5.5%)  6/70 (8.6%)  1/58 (1.7%)  1/38 (2.6%) |  |
| Vascular symptoms  1 year before diagnosis  2 years before diagnosis  3 years before diagnosis  4 years before diagnosis  5 years before diagnosis | 1.095 (0.341-3.516)  0.275 (0.058-1.297)  0.591 (0.196-1.783)  1.080 (0.185-6.292)  N/A* | 0.879  0.103  0.351  0.932 | 5/40 (12.5%)  2/39 (5.1%)  5/36 (13.9%)  2/27 (7.4%)  0/20 (0.0%) | 9/78 (11.5%)  12/73 (16.4%)  15/70 (21.4%)  4/58 (6.9%)  4/38 (10.5%) |  |
| Gait disturbances  1 year before diagnosis  2 years before diagnosis  3 years before diagnosis  4 years before diagnosis  5 years before diagnosis | 0.533 (0.228-1.246)  0.418 (0.143-1.224)  0.353 (0.073-1.706)  0.277 (0.058-1.327)  0.614 (0.060-6.318) | 0.147  0.112  0.195  0.108  0.682 | 10/40 (25.0%)  5/39 (12.8%)  2/36 (5.6%)  2/27 (7.4%)  1/20 (5.0%) | 30/78 (38.5%)  19/73 (26.0%)  10/70 (14.3%)  13/58 (22.4%)  3/38 (7.9%) |  |
| Changes in weight or appetite  1 year before diagnosis  2 years before diagnosis  3 years before diagnosis  4 years before diagnosis  5 years before diagnosis | 1.700 (0.613-4.716)  1.387 (0.409-4.696)  1.625 (0.408-6.467)  1.467 (0.230-9.333)  4.111 (0.348-48.392) | 0.308  0.600  0.491  0.685  0.261 | 8/40 (20.0%)  5/39 (12.8%)  4/36 (11.1%)  2/27 (7.4%)  2/20 (10.0%) | 10/78 (12.8%)  7/73 (9.6%)  5/70 (7.1%)  3/58 (5.2%)  1/38 (2.6%) |  |
| Social indicators  1 year before diagnosis  2 years before diagnosis  3 years before diagnosis  4 years before diagnosis  5 years before diagnosis | 3.818 (1.712-8.515)  2.133 (0.769-5.920)  5.391 (1.302-22.323)  1.687 (0.350-8.130)  9.250 (0.957-89.393) | **0.001**  0.146  **0.020**  0.514  0.055 | 24/40 (60.0%)  9/39 (23.1%)  7/36 (19.4%)  3/27 (11.1%)  4/20 (20.0%) | 22/78 (28.2%)  9/73 (12.3%)  3/70 (4.3%)  4/58 (6.9%)  1/38 (2.6%) |  |
| Daily functioning disturbances  1 year before diagnosis  2 years before diagnosis  3 years before diagnosis  4 years before diagnosis  5 years before diagnosis | 1.178 (0.496-2.799)  1.944 (0.373-10.125)  N/A*  N/A*  1.947 (0.115-32.883) | 0.711  0.430  0.644 | 11/40 (27.5%)  3/39 (7.7%)  0/36 (0.0%)  0/27 (0.0%)  1/20 (5.0%) | 19/78 (24.4%)  3/73 (4.1%)  0/70 (0.0%)  1/58 (1.7%)  1/38 (2.6%) |  |
| *Not applicable, in one or both of the groups zero persons experienced symptoms from this category in this year, therefore no OR has been determined | | | | | |

**Supplemental Result 3. Symptom ORs by year prior to diagnosis for females**

| **Supplemental Table 2.** Odds ratios (OR) with 95% confidence interval (CI) of the symptom categories over the maximum available data before diagnosis and number of females with YOD and females with LOD reporting symptoms over the maximum available data before diagnosis. | | | | |
| --- | --- | --- | --- | --- |
| Symptom category | Odds ratio (95% CI) | *p* | Females with YOD | Females with LOD |
| Cognitive symptoms  1 year before diagnosis  2 years before diagnosis  3 years before diagnosis  4 years before diagnosis  5 years before diagnosis | 0.481 (0.194-1.188)  2.370 (1.068-5.259)  0.844 (0.322-2.212)  1.875 (0.592-5.934)  1.156 (0.112-11.935) | 0.112  **0.034**  0.730  0.285  0.903 | 37/48 (77.1%)  16/43 (37.2%)  7/38 (18.4%)  6/32 (18.8%)  1/16 (6.3%) | 84/96 (87.5%)  19/95 (20.0%)  19/90 (21.1%)  8/73 (11.0%)  3/55 (5.5%) |
| Affective symptoms  1 year before diagnosis  2 years before diagnosis  3 years before diagnosis  4 years before diagnosis  5 years before diagnosis | 2.267 (1.081-4.756)  3.045 (1.281-7.236)  2.229 (0.838-5.929)  1.875 (0.592-5.934)  0.392 (0.045-3.391) | **0.030**  **0.012**  0.108  0.285  0.395 | 20/48 (41.7%)  14/43 (32.6%)  9/38 (23.7%)  6/32 (18.8%)  1/16 (6.3%) | 23/96 (24.0%)  13/95 (13.7%)  11/90 (12.2%)  8/73 (11.0%)  8/55 (14.5%) |
| Behavioral symptoms  1 year before diagnosis  2 years before diagnosis  3 years before diagnosis  4 years before diagnosis  5 years before diagnosis | 0.800 (0.356-1.799)  1.378 (0.467-4.072)  1.194 (0.209-6.815)  0.753 (0.075-7.525)  N/A* | 0.590  0.561  0.841  0.809 | 11/48 (22.9%)  6/43 (14.0%)  2/38 (5.3%)  1/32 (3.1%)  0/16 (0.0%) | 26/96 (27.1%)  10/95 (10.5%)  4/90 (4.4%)  3/73 (4.1%)  1/55 (1.8%) |
| Vascular symptoms  1 year before diagnosis  2 years before diagnosis  3 years before diagnosis  4 years before diagnosis  5 years before diagnosis | 0.702 (0.211-2.334)  0.910 (0.299-2.766)  0.616 (0.162-2.344)  1.155 (0.270-4.938)  0.850 (0.088-8.192) | 0.564  0.868  0.477  0.846  0.888 | 4/48 (8.3%)  5/43 (11.6%)  3/38 (7.9%)  3/32 (9.4%)  1/16 (6.3%) | 11/96 (11.5%)  12/95 (12.6%)  11/90 (12.2%)  6/73 (8.2%)  4/55 (7.3%) |
| Gait disturbances  1 year before diagnosis  2 years before diagnosis  3 years before diagnosis  4 years before diagnosis  5 years before diagnosis | 0.694 (0.333-1.446)  0.437 (0.153-1.244)  0.658 (0.255-1.696)  0.369 (0.099-1.368)  N/A* | 0.329  0.121  0.386  0.136 | 15/48 (31.3%)  5/43 (11.6%)  7/38 (18.4%)  3/32 (9.4%)  0/16 (0.0%) | 38/96 (39.6%)  22/95 (23.2%)  23/90 (25.6%)  16/73 (21.9%)  7/55 (12.7%) |
| Changes in weight or appetite  1 year before diagnosis  2 years before diagnosis  3 years before diagnosis  4 years before diagnosis  5 years before diagnosis | 0.793 (0.305-2.067)  1.125 (0.419-3.023)  2.576 (0.700-9.481)  1.407 (0.315-6.280)  N/A* | 0.636  0.815  0.155  0.655 | 7/48 (14.6%)  7/43 (16.3%)  5/38 (13.2%)  3/32 (9.4%)  0/16 (0.0%) | 17/96 (17.7%)  14/95 (14.7%)  5/90 (5.6%)  5/73 (6.8%)  4/55 (7.3%) |
| Social indicators  1 year before diagnosis  2 years before diagnosis  3 years before diagnosis  4 years before diagnosis  5 years before diagnosis | 4.194 (2.007-8.763)  1.653 (0.583-4.683)  1.088 (0.351-3.377)  8.192 (1.554-43.180)  1.767 (0.150-20.844) | **<0.001**  0.344  0.884  **0.013**  0.651 | 32/48 (66.7%)  7/43 (16.3%)  5/38 (13.2%)  6/32 (18.8%)  1/16 (6.3%) | 31/96 (32.3%)  10/95 (10.5%)  11/90 (12.2%)  2/73 (2.7%)  2/55 (3.6%) |
| Daily functioning disturbances  1 year before diagnosis  2 years before diagnosis  3 years before diagnosis  4 years before diagnosis  5 years before diagnosis | 1.842 (0.836-4.060)  1.952 (0.561-6.786)  2.444 (0.331-18.025)  N/A*  N/A* | 0.130  0.293  0.381 | 15/48 (31.3%)  5/43 (11.6%)  2/38 (5.3%)  0/32 (0.0%)  0/16 (0.0%) | 19/96 (19.8%)  6/95 (6.3%)  2/90 (2.2%)  2/73 (2.7%)  3/55 (5.5%) |
| *Not applicable, in one of the groups zero persons experienced symptoms from this category in this year, therefore no OR has been determined | | | | |

**Supplemental Result 4. Symptom ORs by year prior to diagnosis with age cut-off at 65**

| **Supplemental Table 3.** Odds ratios (OR) with 95% confidence interval (CI) of the symptom categories over the maximum available data before diagnosis, and number of persons with YOD (≤65 years) and persons with LOD (≥66 years) reporting symptoms over the maximum available data before diagnosis. | | | | |  |
| --- | --- | --- | --- | --- | --- |
| Symptom category | Odds ratio (95% CI) | *p* | Persons with YOD | Persons with LOD | |
| Cognitive symptoms  1 year before diagnosis  2 years before diagnosis  3 years before diagnosis  4 years before diagnosis  5 years before diagnosis | 1.217 (0.525-2.819)  1.938 (1.002-3.749)§  0,716 (0.295-1.736)  1.579 (0.609-4.099)  1.435 (0.271-7.588) | 0.647  **0.049**  0.459  0.347  0.671 | 43/51 (84.3%)  20/48 (41.7%)  4/44 (9.1%)  7/37 (18.9%)  2/21 (9.5%) | 174/211 (82.5%)  54/202 (26.7%)  39/190 (20.5%)  20/153 (13.1%)  7/108 (6.5%) | |
| Affective symptoms  1 year before diagnosis  2 years before diagnosis  3 years before diagnosis  4 years before diagnosis  5 years before diagnosis | 1.525 (0.780-2.982)†  1.776 (0.862-3.655)†  0.955 (0.367-2.486)  1.749 (0.668-4.580)  1.386 (0.351-5.471) | 0.218  0.119  0.925  0.255  0.641 | 17/51 (33.3%)  14/48 (29.2%)  6/44 (13.6%)  7/37 (18.9%)  3/21 (14.3%) | 56/211 (26.5%)  39/202 (19.3%)  28/190 (14.7%)  18/153 (11.8%)  12/108 (11.1%) | |
| Behavioral symptoms  1 year before diagnosis  2 years before diagnosis  3 years before diagnosis  4 years before diagnosis  5 years before diagnosis | 1.019 (0.517-2.006)  2.404 (0.998-5.789)  1.601 (0.532-4.818)  N/A*  N/A* | 0.957  0.051  0.402 | 15/51 (29.4%)  9/48 (18.8%)  5/44 (11.4%)  0/37 (0.0%)  0/21 (0.0%) | 61/211 (28.9%)  18/202 (8.9%)  13/190 (6.8%)  6/153 (3.9%)  2/108 (1.9%) | |
| Vascular symptoms  1 year before diagnosis  2 years before diagnosis  3 years before diagnosis  4 years before diagnosis  5 years before diagnosis | 0.939 (0.334-2.640)  1.058 (0.405-2.764)  0.103 (0.014-0.778)§  1.137 (0.299-4.319)  N/A* | 0.906  0.909  **0.028**  0.850 | 5/51 (9.8%)  6/48 (12.5%)  1/44 (2.3%)  3/37 (8.1%)  0/21 (0.0%) | 24/211 (11.4%)  25/202 (12.4%)  33/190 (17.4%)  12/153 (7.8%)  9/108 (8.3%) | |
| Gait disturbances  1 year before diagnosis  2 years before diagnosis  3 years before diagnosis  4 years before diagnosis  5 years before diagnosis | 0.513 (0.253-1.041)  0.598 (0.250-1.429)†  0.724 (0.281-1.868)  0.205 (0.046-0.902)‡  N/A* | 0.065  0.247  0.504  **0.036** | 12/51 (23.5%)  7/48 (14.6%)  6/44 (13.6%)  2/37 (5.4%)  0/21 (0.0%) | 81/211 (38.4%)  44/202 (21.8%)  36/190 (18.9%)  32/153 (20.9%)  11/108 (10.2%) | |
| Changes in weight or appetite  1 year before diagnosis  2 years before diagnosis  3 years before diagnosis  4 years before diagnosis  5 years before diagnosis | 0.641 (0.253-1.621)  1.534 (0.637-3.694)  2.090 (0.743-5.878)  0.819 (0.171-3.923)  N/A* | 0.347  0.340  0.162  0.802 | 6/51 (11.8%)  8/48 (16.7%)  6/44 (13.6%)  2/37 (5.4%)  0/21 (0.0%) | 36/211 (17.1%)  25/202 (12.4%)  13/190 (6.8%)  11/153 (7.2%)  7/108 (6.5%) | |
| Social indicators  1 year before diagnosis  2 years before diagnosis  3 years before diagnosis  4 years before diagnosis  5 years before diagnosis | 4.307 (2.215-8.373)‡  1.505 (0.651-3.481)  2.629 (1.072-6.446)§  4.234 (1.418-12.645)‡  5.712 (1.284-25.411)‡ | **<0.001**  0.339  **0.035**  **0.010**  **0.022** | 35/51 (68.6%)  9/48 (18.8%)  9/44 (20.5%)  7/37 (18.9%)  4/21 (19.0%) | 74/211 (35.1%)  26/202 (12.9%)  17/190 (8.9%)  8/153 (5.2%)  4/108 (3.7%) | |
| Daily functioning disturbances  1 year before diagnosis  2 years before diagnosis  3 years before diagnosis  4 years before diagnosis  5 years before diagnosis | 1.793 (0.915-3.511)  0.918 (0.251-3.359)  1.535 (0.145-16.296)  N/A*  1.781 (0.159-19.989) | 0.089  0.898  0.722  0.640 | 17/51 (33.3%)  3/48 (6.3%)  1/44 (2.3%)  0/37 (0.0%)  1/21 (4.8%) | 47/211 (22.3%)  14/202 (6.9%)  3/190 (1.6%)  3/153 (2.0%)  4/108 (3.7%) | |
| *Not applicable, in one or both of the groups zero persons experienced symptoms from this category in this year, therefore no OR has been determined  ‡ Significant in both the main analysis and the sensitivity analysis (age cut-off at 65).  † Significant in the main analysis only.  § Significant in the sensitivity analysis only. | | | | |  |
